# Crimes without a body: reflections on a case series of online crimes

**DOI:** 10.1093/fsr/owad035

**Published:** 2023-10-04

**Authors:** Cristiano Barbieri, Ignazio Grattagliano, Roberto Catanesi

**Affiliations:** Department of Law, University of Pavia, Pavia, Italy; Department of Education, Psychology and Communication, University of Aldo Moro, Bari, Italy; Section of Clinical Criminology and Forensic Psychiatry, Interdisciplinary Department of Medicine, University of Aldo Moro, Bari, Italy

**Keywords:** body, Web, emotional control perception of the real world, violence

## Abstract

There is a large volume of online crimes. The aim of this work is to reflect on virtual crimes that are apparently different but actually have commonalities. In these cases, the corporeal sphere that mediates interpersonal relationships is absent, and perceptions of the real world and emotional regulation may be altered, which poses the risk of destructive behaviours. From this standpoint, self/hetero-directed aggression is the result of a certain type of transition from the real to the virtual world, where the body either is not involved at all or is experienced in an aberrant manner. In this study, we present three cases that clearly illustrate this concept.

## Introduction

The aim of the present study is to reflect on aspects of apparently different virtual crimes that actually have commonalities. In these cases, because the corporeal sphere that mediates interpersonal relationships is absent or altered, perceptions of the real world and the underlying emotional–affective regulation may be inhibited or impaired, thus posing a risk of destructive behaviours. In fact, self/hetero-directed aggressiveness is the outcome of a sort of “short circuit” arising from the transition from the real to the virtual world and *vice versa*. In this scenario, the human body, the functions of which guarantee the integrity and cohesion of the self, is not involved or is experienced in a pathological manner. In either case, it is no longer a tool enabling affective “I–you” and “I–world” relationships, in which one is simultaneously separate from but at the same time in contact with other people owing to the existence of bodies. Instead, it becomes the tool of a surrogate relationship, in which it seems to exist but is not actually there. There have been various reports of the risk of fragmentation of the self in the virtual world [1] and of the inherent criminogenic potential of this destructured reality [2]. The three cases presented herein clearly illustrate the problems that arise when the body is not involved or is abstracted, as can occur on the Web.

## Case series

### The boy who became a robot

During the COVID-19 pandemic, a 19-year-old girl and a 17-year-old boy, who had been “engaged” for about a year, started to live together at the beginning of the lockdown. The minor left the care of his mother (divorced from his father, who had been living abroad for several years) and his mother’s partner because of conflicts with the latter. The girl left the care of her mother (a widow) because her mother, too, had started to live with a new partner, with whom she had a relationship characterised by conflict. While the girl worked from home, teleworking as a secretary, the boy spent most of his time engaged in role-playing games on a computer. On a single day, he spent over 13 h, without interruption, engaged in an online combat game that involved four players, each of whom lived in a different city. They all played as a robot that must destroy the others to win points and progress to the next level. To eliminate its adversaries, each robot can also sacrifice/lose some parts of its structure. At the end of this extensive online game, the boy was victorious but the girl, on seeing him (as she will later recount) so “absent”, “strange”, and entirely unreactive to her (e.g. not answering her questions), shook the boy and shouted at him to answer her. Suddenly, he punched her in the face, fracturing her jaw, and then ran into the kitchen and thrust his hands into a burning stove, screaming “I’m a C4, I’m a C4”. The burns were so serious that he later had to undergo several surgical procedures. He (i.e. the robot) may have won the game, reaching level C4, because he had eliminated his three adversaries, but he had lost some body parts (both arms) in the process. At the conclusion of the competition, the boy continued to live in the real world as he did in the virtual one, destroying “adversaries” at the cost of losing parts of himself.

### A story of “apparent” love

This tale involves two students, a man and a woman, both of the same age and enrolled at the same faculty. After an engagement lasting ~3 years, the girl broke off the relationship because she had fallen in love with a female study companion, who reciprocated her love. The boy initially expressed aggression in the form of verbal abuse in various chat groups with friends, and he then uploaded films to the Web of himself having sex with his ex-partner. He stated that he had done this in an attempt to recover from the abandonment, which he viewed as a betrayal, albeit that his actions could be construed as pseudo-collegiate exhibitionism. The boy has shown himself to be emotionally immature, with traits connotating significant dependency and impulsivity, and he seemed to have been motivated not only by an impulse toward self-defence, characterised by an ostentatious display of his own virility, but above all by a desire for revenge and a need to “get back” at the girl. He had quickly discovered, almost by chance, the affair between the two women and had acted in response to feelings of a double betrayal, as an exclusive partner but especially as a man. Penal actions taken by the victim were later abandoned owing to mediation efforts between the parties supported by their respective lawyers. The girl later left her companion for another female colleague. The boy moved to another city and started a relationship with a distant relative of his ex-partner.

### A case of cybersex addiction

The couple in this case consisted of a 26-year-old man (employed as a computer technician) and a 25-year-old woman (employed as an accountant). Neither of them had a history of any previous significant romantic relationship. They had met at a local parish meeting. After an engagement lasting ~2 years, during which, by mutual choice, they were only partially intimate (with rare episodes of reciprocal masturbation), they got married. Afterwards, they were unable to consummate the marriage because of serious erectile dysfunction that the man attributed to intense stress at work. He limited investigations to one visit by a urologist, followed by one from an andrologist, along with blood tests and hormonal and angiological assessments that excluded any organic cause. However, as time passed, he spent progressively less time with his wife, staying in his laboratory on the pretext that he needed to catch up on work backlogs. A year and a half after the wedding, the woman discovered that he actually spent endless hours on a computer watching pornographic films and compulsively masturbating. When she managed to get him to consult a psychiatrist, a diagnosis of cybersex addiction accompanying anxiety–depression syndrome was made, and supportive and symptomatic therapy was prescribed (an anxiolytic and an antidepressant). The man agreed to take the drugs but refused to see a psychotherapist. When his wife presented him with an either/or choice (“Either you get proper help or I leave you”), he reacted by carrying out serious acts of self-harm (he ingested powder rat poison and drank a bottle of gin), which he later ascribed to a desire to “solve the problem once and for all after so many years”. He survived owing to timely admission to the hospital. Two months after this episode the woman left him, declined any further communication, and went to live abroad.

## Critical reflections

The first case demonstrates what can happen when no body is involved in a crime, as occurs on the Web, but rather only an abstracted version or simulacre of a body. The second and third cases confirm that, on the Web, users can easily be manipulated in cases of both instrumental and dysfunctional relationships. In fact, on the Web, the body can become the object of instrumental manipulation and lose its power to mediate perceptions, integrate cognition with emotion, and regulate emotional–affective relationships and aggressiveness. Inevitably, this has negative consequences regarding the ability to accurately appraise reality (as in the first case) and adequately interact with the Self (as in the second and third cases). In fact, in the first case the individual became, to all intents and purposes, a robot that wins but ultimately destroys itself during the process of overcoming its adversaries, first in the virtual world and then in the real world. In the second case, the individual did not take account of the consequences of his actions (publishing pornographic films featuring sexual intercourse with his ex-partner on the internet), nor did he have insight into his own motivations or the implications or significance of his actions. Therefore, his desire for revenge, triggered by a narcissistic injury, manifested as irresponsible actions construable as collegiate exhibitionism. His feelings were triggered by a traumatic life experience (the discovery of a double betrayal, as the elective partner and as a man), initially manifesting as a feeling of abandonment. In the third case, the individual developed a severe somatic sexual disturbance, although this originated from a psychological disturbance that was maintained and amplified by the internet. The body became the object of a serious psychiatric disorder, leading to a form of dependency fostered through his relationship and interactions with the internet. In turn, this motivated self-directed aggression in a completely aberrant attempt to solve the problem. We reflect on specific aspects of these cases below.

### Cognitive distortions are correlated with excessive use of online tools, which undermines normal interactions with the real world

The internet has changed our behaviours, our ways of thinking and perceptions of reality, and the way we see ourselves and our relations with the world and the rest of humanity. The most radical changes in human existence seem to relate to ideas regarding the body and sexuality arising in the 21st century. In particular, the concept of cybersex has developed rapidly in recent years, sometimes degenerating into true techno-mediated crime and feeding a specific type of addiction. In fact, since the advent of the World Wide Web, devices and applications such as personal computers, smartphones, social networks, informatic platforms have not only emerged as useful tools for work, study, and obtaining up-to-date information, but have also served as intermediaries in interpersonal communication. These tools are sufficiently powerful to affect the timing and forms of individual expression, transforming the way people perceive life, think, and act [3, 4]. Indeed, social networks constitute a “nowhere” space within which every user can build a virtual identity and share aspects of their life with others possessing similar views. Moreover, the decisional processes associated with the sharing of information are affected by the intrinsic characteristics of those who take part, where the cornerstone is the complex and reciprocal interactions between the individual and the context. In this respect, it should be borne in mind that emotions, as internal mental states of varying intensity and duration, represent the expression of certain types of input [5, 6]. As such, they play a fundamental role in the way in which an individual interacts with environmental stimuli according to cognitive evaluations and subsequent actions [7, 8]. The formation of “first impressions” can lead to accurate or inaccurate interpretations of new information [9–11]. People often have difficulties in interpreting and processing information when it contrasts with their preexisting cognitive structures, even when subsequent feedback reveals that such information is false. Because sharing is not the product of a single action but rather a process that is affected by multiple variables [12], when there are excessive and anomalous interactions between the real and virtual worlds, there is a high risk of confusion between fantasy and reality. This can have extremely serious consequences not only for behaviour on the internet but also in the real world.

### Sexuality online

Online, even timid people who are afraid of their sexuality, impelled perhaps simply by curiosity, may “take a look at a world that was unknown to them until just before” [3]. However, the initial curiosity can lead to the discovery that, in this new world, they can live not only in an alternative manner but also satisfy their sexual needs, triggering a desire to go further. The opportunity to experience explicit content afforded by anonymity can not only lead the individual to discover, and freely indulge in, forms of sexual excitement they knew nothing about previously (because they had previously avoided such forms), but also to move away from real intimate relationships. Cybersex is thus an erotic behaviour produced by the “completion of a shared representation”, and mediated by online contacts, which can take on peculiar characteristics that are quite different from normal masturbation, being founded on an internal self-created and -directed representation of a possible sexual interaction where the fantasy does not need to take into account the feelings and needs of other individuals [4]. However, although for some cybersex remains an occasional indulgence, for others it can develop into a true pathological addiction with serious negative consequences in such areas as the social, relational, work, and economic domains [13–16]. It can also manifest as “revenge porn”, in which explicit sexual material is disseminated without the consent of the person depicted in the interests of revenge, i.e. “getting one back” and punishing the victim. The consequences of this can be serious, ranging from job loss to transferring to another branch to avoid social stigma, humiliation and harassment, various types of psycho-reactive disorders, and even suicide [17–19]. Techno-mediated experiences may transform into a fundamental need, or addiction, to which everything else is seen as secondary and thus must be sacrificed [20]. The central core of many perverse activities is a deep fear of losing one’s identity [21]. In fact, some sexual practices become a tool to deal with a sense of impending death and the fear of disintegration of the self. Therefore, even if analysis of the individual’s internal world is useful to gain an understanding of their intrapsychic and interpersonal functioning, it is also essential to evaluate their level of understanding of and relationship with objective reality, as mediated by their own body and sexuality [22]. Sometimes, an individual may try to satisfy their unmet needs in the virtual reality environment: the initial search for intimate relations to alleviate stress may give rise to the illusion that, in this way, they can control negative and painful feelings in real life. However, this actually raises the risk of progression to perverse sexual behaviours to which all else are subordinated.

### Addiction

While on one hand, it is claimed that all forms of addiction have affective dependency in common [23–25], on the other hand, it is asserted that internet addiction includes at least four distinct types of online dependency: cyber sexual addiction, cyber relational addiction, net compulsion, and information overload. There is also some doubt as to whether the diagnoses of “sexual dependency” and “hypersexual disorder” in fact refer to the same problem [26] because both are characterised by a spectrum of behaviours ranging from anankastic to anaclitic. This spectrum also covers quantitative (hypersexual disorder) and qualitative (the severity of compulsive–impulsive disorders) aspects [27]. Regardless of the classification or cause, for the World Health Organization, the abuse of digital technologies is always a public health concern [28] owing to the potential negative effects thereof. Moreover, research on this issue has pointed to the existence of many internet-related disorders, ranging from anxiety–depressive syndromes to sexual disorders and from socio-relational problems to familial, vocational, and financial issues [29–35].

## Conclusions

The progression from feelings of omnipotence to a sense of dominion, and ultimately desperation, seen in techno-mediated relationships clearly exemplifies the paradox involved in relationships between the human mind and advanced information technologies. In fact, a certain type of internet use is marked not only by its accessibility, economic nature, and rapidity of interaction but also by the potential to arbitrarily alter the personal identity and psychic integrity of the individual through the “elimination” of the body or its transformation into an virtual object on the internet. This poses an extremely high risk of a progressive and ultimately total loss of control of the techno-mediated experience, in turn giving rise to a risk of committing or being the target of a genuine crime [36]. The internet has radically changed the speed and nature of communication, which has become a bodiless or body-transforming experience. Moreover, the ease of information circulation has not only led to the spread of constructive and educational content but also to the dissemination of content that is dangerous and destructive. In fact, the internet has given rise to new clinical disorders and crimes [37–39]. As illustrated by the cases described herein, the danger of becoming a perpetrator and/or victim of crimes appears to be extremely high in the context of new forms of collective violence and collective victimisation [40].

## Authors' contributions

Ignazio Grattagliano, Cristiano Barbieri and Roberto Catanesi contributed to the data curation and analysis, and article writing. Cristiano Barbieri and Ignazio Grattagliano contributed to the conception of the project. Roberto Catanesi participated in the conception of the project, supervised the study and edited the article.

## Compliance with ethical standards

Ethical approval for the study was obtained from the Ethics Committee of the University of Aldo Moro. Written informed consent was obtained from all individual participants involved in the study.

## Disclosure statement

No potential conflict of interest was reported by authors.
